# Perioperative the BTLA inhibitor (tifcemalimab) combined with toripalimab and chemotherapy for resectable locally advanced thoracic esophageal squamous cell carcinoma trial (BT-NICE trial): a prospective, single-arm, exploratory study

**DOI:** 10.3389/fimmu.2025.1542877

**Published:** 2025-04-10

**Authors:** Chengzhi Ding, Yahao Zhang, Tian Xia, Jiwei Li, Wenjian Yao, Quan Zhang, Zhijun Han, Jianjun Wang, Zhikun Cao, Jinlong Hu, Li Wei

**Affiliations:** ^1^ Department of Thoracic Surgery, Henan Provincial People’s Hospital; Zhengzhou University People’s Hospital, Zhengzhou, China; ^2^ Department of Oncology, Henan Provincial People’s Hospital; Zhengzhou University People’s Hospital, Zhengzhou, China

**Keywords:** esophageal squamous cell carcinoma, the BTLA inhibitor, toripalimab, esophagectomy, immunotherapy

## Abstract

**Background:**

The treatment of cancer has brought about a paradigm shift with the introduction of immune checkpoint blockade (ICB) therapy, which is mostly dependent on inhibiting PD-1/PD-L1 and CTLA-4. However, recent studies have shown limited efficacy of this treatment in esophageal squamous cell carcinoma (ESCC). Preliminary studies have found that tifcemalimab (the world’s first anti-BTLA blocking monoclonal antibody) combined with toripalimab (PD-1) and chemotherapy has shown favorable safety and efficacy in several solid cancers. This study aimed to evaluate the safety and efficacy of neoadjuvant tifcemalimab combined with toripalimab and chemotherapy following esophagectomy for resectable ESCC, and the association of adjuvant immunotherapy with improved survival outcomes.

**Methods:**

Patients with pathologically confirmed cT1b-3N1-3M0 or cT2-3N0M0 thoracic ESCC were treated with neoadjuvant tifcemalimab (200mg, iv, d1) in combination with toripalimab (240mg, iv, d1) and chemotherapy (paclitaxel 135-175 mg/m^2^, d1 + cisplatin 75 mg/m^2^, d1) every 3 weeks for 2 cycles. Patients undergoing esophagectomy with pathological complete response (pCR) were administered up to 15 cycles of adjuvant tifcemalimab (200 mg) and toripalimab (240 mg), whereas patients without pCR received tifcemalimab in combination with toripalimab and adjuvant chemotherapy for 2 cycles, followed by tifcemalimab in combination with toripalimab immunotherapy up to 13 cycles. The patient with incomplete resection was decided to receive radiotherapy after a multidisciplinary consultation. The primary endpoint of this study was the pCR rate. The secondary endpoints include major pathological response rate (MPR), objective response rate (ORR), disease control rate (DCR), adverse events, R0 resection rate, event-free survival (EFS), and overall survival (OS).

**Discussion:**

The Ethics Committee of Henan Provincial People’s Hospital has approved the protocol (No 2024-132-03). This study is the world’s first prospective clinical trial to evaluate the safety and efficacy of the BTLA inhibitor in combination with PD-1 and chemotherapy as neoadjuvant/adjuvant therapy for locally advanced thoracic ESCC. We predicted that perioperative combination immunotherapy as a potentially preferred and effective treatment strategy may lead to better survival outcomes.

## 1 Background

In the chemotherapy era, the role of surgery for locally advanced thoracic esophageal squamous cell carcinoma (ESCC) is controversial with survival benefits limited by lymphatic metastasis and incomplete resection from esophagectomy (1–3). Recently, the potential advantages of immune checkpoint blockade therapies in various solid tumors have revolutionized the treatment landscape of esophageal cancer (4–6). Moreover, immunochemotherapy has become the cornerstone of the treatment of esophageal cancer beyond traditional chemoradiotherapy (CRT) (7, 8). However, preliminary results show that the tumor response rate of PD-1 or CTLA-4 blocking antibodies was no more than 20-30%, and about 30-40% with esophageal cancer exhibit gross immunotherapy resistance (9). Therefore, there is an urgent need to discover new potential targets to expand immune receptors and synergistically modulate immune responses to enhance immunotherapy efficacy. Expanding the portfolio of targets with novel checkpoints that collectively improve the effects of the immune blockade has the potential to benefit more patients and maybe a new approach over the past few years.

When combined with toripalimab (anti-PD-1), Tifcemalimab—the world’s first-in-class humanized IgG4 monoclonal antibody targeting B and T lymphocyte attenuator (BTLA)—has demonstrated prior anti-tumor affects in pretreated patients with advance solid tumors (10). It belongs to the CD28 receptor family and possesses a single IgSF V extracellular domain that is sequence identical to members from the CD28 family, including CTLA-4 and PD-1. Its receptor recognizes and binds to the herpesvirus entry mediator (HVEM), which belongs to the TNF receptor family. Based on the preclinical study HVEM binding to BTLA prevents T- and B-cells from being activated, proliferation, and cytokine generation (10). Tifcemalimab with toripalimab displayed an excellent safety profile and encouraged antitumor efficacy in patients with non-small cell lung cancer (NSCLC), melanoma, renal cell carcinoma (RCC), and lymphoma in a phase Ia/Ib research carried out at Wisconsin Carbone Cancer Center (11). Meanwhile, in a phase, Ib/II open-label trial, tifcemalimab combined with toripalimab and chemotherapy demonstrated encouraged objective response rates (ORR) and a manageable safety profile as the initial line of treatment for patients with extensive-stage small lung cancer (ES-SCLC) (12). According to the Checkmate 577 research, patients who failed to show pCR to neoadjuvant chemoradiotherapy had a substantially longer disease-free survival (DFS) when postoperative adjuvant nivolumab was used (13). Therefore, whether the combination of adjuvant tifcemalimab and toripalimab provides additional benefits after surgery is an unanswered question.

The BT-NICE trial is the first exploratory official report to evaluate the safety and effectiveness of neoadjuvant anti-BTLA and PD-1 combined IO-IO strategies with chemotherapy and adjuvant IO-IO strategies in the treatment of ESCC, which poses a novel challenge for the current treatment strategies. This trial was registered at ClinicalTrials. Gov (NCT 06588335).

## 2 Methods/design

### 2.1 Study design

The BT-NICE trial was a prospective, single-center, exploratory phase II research designed to evaluate the safety and effectiveness of adjuvant tifcemalimab combined with toripalimab as postoperative immunotherapy might improve survival for patients with locally advanced thoracic ESCC, and the combination of neoadjuvant tifcemalimab with toripalimab and chemotherapy treatment. The primary endpoint was the pCR rate in all protocol patients. The lack of staying tumor cells was defined as pCR in the resected lymph nodes and the primary site of the surgical tissues.

The following were the secondary endpoints: adverse events, event-free survival (EFS), overall survival (OS), major pathological response (MPR) rate, disease control rate (DCR), objective response rate (ORR), and R0 resection rate. Less than 10% residual tumor main tumor in the resected and all resected lymph nodes in the samples acquired following neoadjuvant treatment was considered the MPR rate. The proportion of patients who had a primary tumor volume decrease of 30% and were able to sustain it for more than four weeks, as determined by the investigator using RECIST v1.1, was known as the objective response rate (ORR). This is equivalent to adding the proportions of complete remission (CR) and partial remission (PR). DCR is the ratio of the number of patients with disease control [CR, PR, and stable disease (SD)] to the total number of evaluable patients. Rate of Adverse Events Associated with Treatment as determined by CTCAE v5.0. During perioperative care, all adverse events, including surgical morbidity and death, are documented. For surgical morbidity, we referenced the International Consensus by the Esophagectomy Complications Consensus Group (ECCG) (14), which is a crucial indicator for evaluating the effectiveness and safety of surgery.

**Figure 1:**
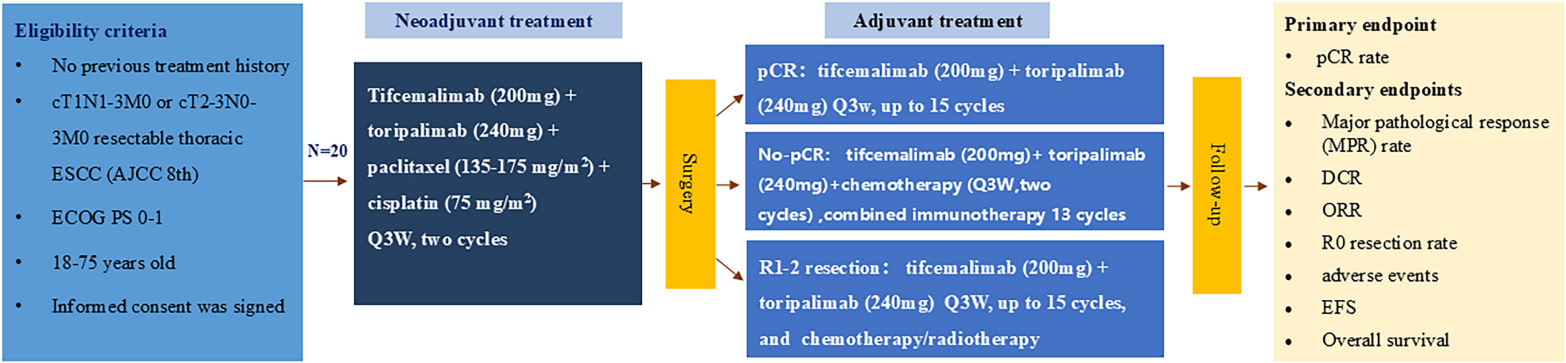
Flaw chart.

R0 resection is defined as the removal of esophageal and lymph node lesions with negative margins and anastomosis without tumor residue. The time interval between randomization and the onset of illness progression, therapy cessation for any cause, or death was designated as EFS. In **Figure 1**, the trial flow chart is displayed. The projected end date of the research is August 31, 2026, with an initial start date of September 5, 2024.

### 2.2 Study population

The Henan Provincial People’s Hospital Ethics Committee authorized this study in August 2024 (No. 2024-132-03). The lead investigator selected patients with locally advanced ESCC according to inclusion/exclusion criteria. Patient recruitment began in September 2024 and is still in the recruitment phase.

### 2.3 Eligibility criteria

#### 2.3.1 Inclusion criteria

1. The patients gave their approval to participate in the trial and to comply with the follow-up visits by signing an informed consent form;
2. Age 18-75 years old;
3. Histologically confirmed clinical stage of locally advanced (cT1N1-3M0 or cT2-3N0-3M0) thoracic ESCC (8th UICC-TNM staging);
4. Enhanced CT of the neck shows no suspicious metastatic lymph nodes (excluding lymph nodes in the esophageal cancer area), and imaging examination shows no distant metastasis;
5. ECOG: 0~1 score;
6. Expected to achieve R0 resection;
7. No prior antitumor therapy for esophageal cancer and no other tumor therapy;
8. Presence of measurable lesions and lymph nodes (according to RECIST 1.1 criteria);
9. Application of appropriate organs and hematopoiesis in bone marrow. The following requirements must be satisfied: Absolute neutrophil count (ANC) ≥1.5×10^9^/L; Hemoglobin ≥90g/dL; Platelets ≥100×10^9^/L; Serum albumin ≥2.8g/dL; Total bilirubin ≤1.5×ULN, ALT, AST and/or AKP ≤2.5×ULN; Serum creatinine ≤1.5×ULN or creatinine clearance ≥60mL/min (calculated by Cockcroft-Gault formula); International Normalized Ratio (INR) and Activated Partial Thromboplastin Time (APTT) ≤1.5×ULN (those treated with stable doses of anticoagulants, such as low molecular weight heparin or warfarin, and whose INR is within the expected therapeutic range of the anticoagulants);
10. For female patients who are potentially fertile, the desire to abstain from pregnancy before enrollment, throughout the research period, and for six months following the final medication dosage.

#### 2.3.2 Exclusion criteria

1. Patients at high risk of bleeding, fistula, or perforation.
2. Individuals suffering from serious malfunctions related to the hematopoietic system, endocrine system, liver, kidney, heart, or cachexia.
3. Patients had undergone major surgery (excluding diagnostic surgery) or major trauma and had received anticancer drugs and vaccines within 4 weeks before treatment.
4. Patients with electrolyte abnormalities >Grade 1 that cannot be corrected before first study drug administration.
5. Individuals with any history of or current autoimmune illness, excluding vitiligo and allergies/asthma that do not require treatment in adulthood.
6. Immunocompromised patients, such as those with HIV, acquired or congenital immune deficiencies, organ transplant recipients, or recipients of allogeneic bone marrow transplants.
7. Individuals with uncontrolled heart conditions, such as unstable angina pectoris, heart failure (NYHA Class II or above), myocardial infarction within one year, or clinically severe arrhythmias needing medical attention.
8. Individuals who had a significant illness (CTCAE >Grade 2) four weeks before the first medication was administered for the research.
9. Individuals with detectable HCV-RNA and positive HCV antibody or those with active hepatitis C (HBV DNA ≥ 2000 IU/mL or 104 copies/mL).
10. Patients with known hypersensitivity, allergy, or contraindication to JS004, toripalimab, or any of their formulation components.
11. Patients with any other malignancy, except low-risk or completely cured malignancies.
12. Pregnant, lactating, or unwilling to use contraception.
13. Additional criteria that might result in the research being stopped include individuals who are at a high risk of recurrence, significant problems requiring medication, severe laboratory abnormalities, and family/social circumstances that could compromise patient safety or data collection.

### 2.4 Interventions

#### 2.4.1 Preoperative treatment

Patients received neoadjuvant tifcemalimab (200mg, iv, d1) combined with toripalimab (240mg, iv, d1) plus chemotherapy (paclitaxel 135-175 mg/m^2^ + cisplatin 75 mg/m^2^, d1) every 3 weeks for 2 cycles. Reductions of tifcemalimab and toripalimab were not permitted unless serious immune-related adverse effects led to discontinuation, but a 30% dose reduction of chemotherapy was allowed except for severe laboratory abnormalities (CTCAE > Grade 2). Treatment should be interrupted or delayed if a serious adverse event occurs. Continue treatment when recovery meets the criteria for re-treatment.

#### 2.4.2 Surgery

The patients were reviewed by the researchers using RECIST 1.1 following 4-6 weeks of neoadjuvant treatment. Patients classified as having clinically stable disease (cSD), clinical partial remission (cPR), and clinical complete remission (cCR) underwent surgery, while those classified as having progressing diseases underwent further therapies following a multidisciplinary consultation.

The patient underwent thoracic-laparoscopic esophagectomy with McKeown procedure, which consisted of right open thoracotomy or robot-assisted two-field lymph node dissection.

Using the McKeown procedure, patients undergo thoracoscopy in addition to laparoscopy-aided esophagectomy; open or robotically assisted procedures may also be engaged. Transhiatal or left thoracotomy esophagectomy was excluded due to difficulties in upper mediastinal lymph node dissection. Complications after surgery will be recorded utilizing the Clavien-Dindo classification in the case report form.

#### 2.4.3 Adjuvant therapy

After 4-8weeks postoperatively, patients with pCR will receive adjuvant therapy with tifcemalimab (200 mg) and toripalimab (240 mg) every 3 weeks up to 15 cycles, while patients without pCR will receive tifcemalimab (200 mg) combined with toripalimab (240 mg) and chemotherapy (paclitaxel 135-175 mg/m^2^ + cisplatin 75 mg/m^2^) every three weeks for two cycles, follow by maintenance therapy with tifcemalimab (200 mg) in combined with toripalimab (240 mg) up to 13 cycles. The patient with incomplete resection was decided to receive radiotherapy after a multidisciplinary consultation.

### 2.5 Safety analysis

The primary metrics for safety-related analyses were adverse events (treatment-related and immune-related) and surgical-related complications, which were evaluated using the National Cancer Institute Common Terminology Criteria for Adverse Events, version 5.0 (CTCAE5.0) (15). Grade 3 to 5 TRAEs were defined as serious adverse events (SAEs), for which appropriate protective measures should be adopted or treatment should be terminated and the SAE Report Form should be completed within 24 hours.

### 2.6 Pathologic examination

The pathology reports were evaluated by two professional pathologists, including histological type, depth of tumor invasion and percentage of residual tumor, peripheral and upper margins of circumferential incision, peripheral lymph node involvement, and tumor regression grading (TRG) (16).

### 2.7 Follow-up

The first follow-up is performed 3 months after surgery, even though postoperative chemotherapy was still being administered at that time. After that, follow-ups were scheduled every three months for the first year of therapy and every six months for the second year until the conclusion of the five-year term or death. A physical examination, routine laboratory testing, and an improved CT scan of the upper abdomen and thorax are all part of the comprehensive evaluation program. Patients with documented signs of recurrence may also undergo additional tumor evaluation during treatment. However, no other cytotoxic drugs may be used during the follow-up period.

### 2.8 Statistics

Using the EXACT method, the width of the confidence interval was assessed assuming that a pCR rate of 30% could be achieved in the treatment group and that enrolling 20 subjects would generate a bilateral 95% confidence interval with a width equal to 0.396. The first stage was supposed to comprise seventeen patients. A total of twenty patients were needed, accounting for a twenty percent drop-out rate. We considered 40% to be the lowest allowable MPR rate. Neoadjuvant treatment was considered unsuccessful if the MPR rate was less than 40%. The neoadjuvant regimen was deemed unsuccessful and the experiment was stopped if < 3 patients experienced an MPR during the first stage. We considered 40% to be the lowest allowable MPR rate. If it was below 40%, the treatment was considered ineffective. If less than 4 out of 10 patients in the first phase show an MPR, the trial will be terminated.

The results of this study were mainly based on statistical descriptive methods. Measures were listed as mean, standard deviation, median, maximum, and minimum, and counts and ranks were listed as frequency (constitutive ratio), rate, and confidence interval. Efficacy analysis: for efficacy endpoints such as pCR, and ORR. Their 95% confidence intervals were estimated using the Clopper Pearson technique. The 95% confidence intervals for the median EFS, median OS, and their representative overall were estimated using the Kaplan-Meier technique, and survival graphs were created. Safety analysis: the incidence of adverse events and adverse reactions were examined using descriptive statistical analysis.

### 2.9 Bio-sampling program

**Figure 2:**
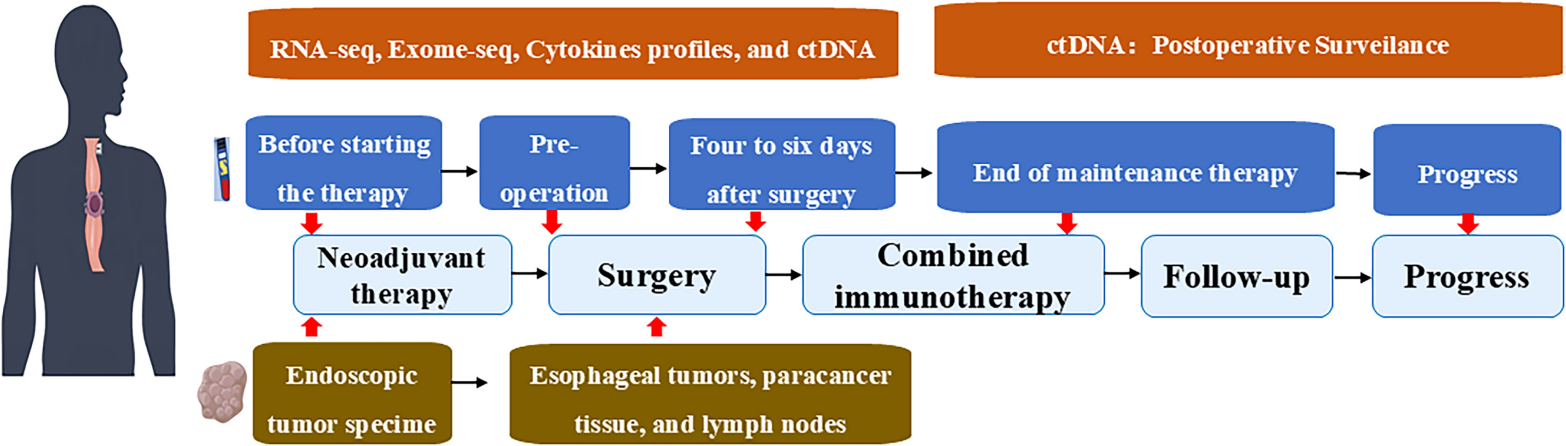
Study design.

The study will also sign consent forms that include a translational bio-sampling procedure, which will become a platform for future research projects. The prospective biological specimen collection plan included blood, tumor, para-cancerous tissues, and lymph nodes for data analysis such as RNAseq analysis, exome analysis, cytokine profiling, and ctDNA. According to the study design, samples were collected at five eligible time points: pre-treatment, pre-operation, four to six days after surgery, at the end of maintenance therapy, and at the time of progress (Study design is depicted in **Figure 2**).

## 3 Discussion

This is the first study in the world to report the role of the BTLA inhibitor in combination with anti-PD-1 and chemotherapy as neoadjuvant treatment for locally advanced ESCC and as postoperative adjuvant IO-IO combination immunotherapy.

Currently, the superior treatment for locally advanced ESCC is simultaneous chemoradiotherapy followed by esophagectomy, with complete pathologic response rates ranging between 43.2% and 49% (17). The long-term results from the CROSS and NEOCRTEC5010 trials validated local recurrence and distant metastases within 2 years in 27%-32.3% of patients, especially in non-pCR patients, suggesting that nCRT combined with surgery is insufficient to improve long-term survival (16, 18). Neoadjuvant immunotherapy has been shown in recent years to bestow systemic anti-tumor immunity and durable anti-tumor immune responses, which may offer these patients additional advantages. In the NICE study (19), KEEP-G 03 study (20), and TD-NICE study (21) in neoadjuvant immunotherapy combination chemotherapy treatment in patients with locally advanced ESCC the pCR rates were 39.2%, 20.0%, and 50.0%, respectively. Compared with the pCR rates of 49% (CROSS study), and 43.2% (NEOCRTEC5010 study) for preoperative induction radiotherapy, respectively, it seems that teraplizumab in TD-NICE has a higher pCR rate. For patients with R0 resection, postoperative adjuvant immunotherapy is crucial. In the CheckMate 577 trial (13), the median disease-free survival of those on nivolumab adjuvant therapy was 22.4 months, compared to 11.0 months for placebo recipients (hazard ratio 0.69), showing it can prolong disease-free survival, reduce recurrence/death risk and improve prognosis. In the ESCORT - NEO trial (8), the Cam + nab - TP and Cam + TP groups had higher pCR rates of 28.0% and 15.4% respectively, compared with 4.7% in the chemotherapy-alone group, which demonstrated the overall benefits of perioperative immunotherapy. Furthermore, the PALACE-1 study demonstrated that PD-1 inhibitors combined with preoperative chemoradiotherapy resulted through pCR 55.6% of resected tumors; even so, 65% of patients had grade III adverse events (AEs), including one death (22). There is evidence suggests that achieving a higher pCR is accompanied by a higher risk of chemoradiation and that increasing immune-related antitumor effects to achieve a higher pCR may be a low-risk and safe approach.

The BTLA inhibitor, similar to PD-1 and CTLA-4, belongs to the CD28 immunoglobulin superfamily with similar structural features (23). While PD-1 is extensively expressed on activated T cells, B cells, and myeloid cells, BTLA is primarily found on T cells, B cells, and dendritic cells (DCs). Sometimes tumor-specific CD8+ T lymphocytes with a largely dysfunctional phenotype co-express BTLA and PD-1 (24). The immunosuppressive signaling mechanism generated by the binding of BTLA to HVEM inhibits T-cell and B-cell activation primarily by attracting the tyrosine phosphatases SHP-1 and SHP-2, and may simultaneously promote the survival of T-cells under specific conditions. It inhibits T and B cell activation and proliferation as well as cytokine production and alleviates the dilemma of T cell immune depletion due to sustained high PD-1 expression (10). Hence, the combined blockade of BTLA and PD-1 enhances the effect of immunotherapy.

To the best of our knowledge, BTLA-HVEM’s involvement in real-world human tumors is still mostly unclear. Derre et al. showed that BTLA binding to HVEM receptors on melanoma cells inhibits IFN-g secretion and tumor-specific T cell proliferation, demonstrating that HVEM-BTLA inhibitory interactions can play a role in anti-tumor immune evasion (25). Malissen N et al. also found that HVEM was expressed in 65%-98.3% of melanomas by immunohistochemistry, BTLA was expressed in CD8+ tumor-infiltrating Lymphocytes (TILs) in 11.1% of patients, and HVEM was more widely expressed than PD-L1 by flow cytometry (43% vs 1.6%) (26).

There is currently little information available on this immunotherapeutic agent’s clinical effectiveness. A phase Ib/IIA open-label study (NCT05664971) with 37 patients with ES-SCLC as first-line treatment with tifcemalimab combined with toripalimab and chemotherapy demonstrated encouraging clinical remission rates with a manageable safety profile (32 partial remissions, 5 stable disease, ORR 86.5% and DCR 100%, remission continued in 94.6% of the patients, and the median length of remission was not yet) (12). Based on a preliminary study, patients exhibiting positive co-expression of PD-L1 or HVEM expression demonstrated 100% ORR, suggesting that this might be a promising biomarker. Meanwhile, in an, I study (NCT 04137900) using tifcemalimab combination with the PD-1 blocking toripalimab in patients with advanced malignancies, with a median follow-up of 11.4 weeks (11). After receiving a median of four previous lines of treatment, all 57 evaluable patients saw one full response (lymphoma), six partial responses (two each for melanoma, renal cell carcinoma, non-small cell lung cancer, and urothelial carcinoma), and 17 stable illnesses. It’s interesting to note that there was a positive trend association between clinical response and high expression of HVEM. In conclusion, more studies on the BTLA/HVEM axis as a potential target for immunotherapy are encouraged by the early clinical success in solid tumors and lymphomas.

In summary, the purpose of this BT-NICE trial was to determine if adjuvant tifcemalimab combined with toripalimab as a postoperative immunotherapy would improve survival benefits for patients with locally advanced thoracic ESCC, and whether neoadjuvant tifcemalimab in collaboration with toripalimab plus chemotherapeutic treatment would offer adequate safety and effectiveness. The trial helps to answer the following questions: first, whether neoadjuvant therapy with BTLA inhibitors in combination with PD-l and chemotherapy (TP regimen) better improves tumor downstaging and achieves higher pCR rates; second, whether IO-IO immunotherapy results in more associated immune adverse effects; and third, whether BTLA inhibitors and PD-l adjuvant therapy provide non-CR and pCR patients with additional dual survival benefit. Speculation indicated that perioperative IO-IO combination immunotherapy may be a new pattern regimen for immunotherapy of esophageal cancer, which can improve survival by enhanced immune-combination effects.
